# Bank vole alarm pheromone chemistry and effects in the field

**DOI:** 10.1007/s00442-021-04977-w

**Published:** 2021-06-25

**Authors:** Thorbjörn Sievert, Hannu Ylönen, James D. Blande, Amélie Saunier, Dave van der Hulst, Olga Ylönen, Marko Haapakoski

**Affiliations:** 1 Department of Biological and Environmental Science, Konnevesi Research Station, University of Jyväskylä, P.O. Box 35, 40014 Jyväskylä, Finland; 2 Department of Environmental and Biological Sciences, University of Eastern Finland, P.O. Box 1627, 70211 Kuopio, Finland; 3 Environmental Sciences Department, Resource Ecology Group, Wageningen University, 6700 AA Wageningen, Netherlands

**Keywords:** Bank vole, Alarm pheromone, Mammalian body odour, Predator–prey interactions

## Abstract

Chemical communication plays an important role in mammalian life history decisions. Animals send and receive information based on body odour secretions. Odour cues provide important social information on identity, kinship, sex, group membership or genetic quality. Recent findings show, that rodents alarm their conspecifics with danger-dependent body odours after encountering a predator. In this study, we aim to identify the chemistry of alarm pheromones (AP) in the bank vole, a common boreal rodent. Furthermore, the vole foraging efficiency under perceived fear was measured in a set of field experiments in large outdoor enclosures. During the analysis of bank vole odour by gas chromatography–mass spectrometry, we identified that 1-octanol, 2-octanone, and one unknown compound as the most likely candidates to function as alarm signals. These compounds were independent of the vole’s sex. In a field experiment, voles were foraging less, i.e. they were more afraid in the AP odour foraging trays during the first day, as the odour was fresh, than in the second day. This verified the short lasting effect of volatile APs. Our results clarified the chemistry of alarming body odour compounds in mammals, and enhanced our understanding of the ecological role of AP and chemical communication in mammals.

**Supplementary Information:**

The online version contains supplementary material available at 10.1007/s00442-021-04977-w.

## Introduction

Predator–prey interactions are among the strongest drivers of evolution (Abrams 1986, 2000; Yoshida et al. 2003). In the context of an evolutionary arms race, early recognition of predation risk by prey is essential for prey survival and fitness. Cues of increased predation risk range from very reliable cues like sighting of a predator or its direct attack (Blumstein et al. 2000; Van der Veen 2002), to more general and less accurate ones like signs or markings of predator revealing its presence or visit in vicinity. These signs include odorous faeces or other scent cues (Kats and Dill 1998). However, these cues do not necessarily have to originate from the predator, as the other option for information on predator are cues carried by conspecific prey, which often can even be more reliable than a mere predator odour (Blumstein et al. 2000; Randler 2006; MacLean and Bonter 2013).

After perceiving increased predation risk, multiple mechanisms and adaptations by prey animals are possible, from simple immediate behavioural responses to long-term physiological or even intergenerational adaptations (Abrams 2000). Anti-predatory behaviours employed in prey range from simple avoidance of high-risk areas (Ferrero et al. 2011; Clinchy et al. 2013; Pérez-Gómez et al. 2015) and freezing to decrease detectability (Wallace and Rosen 2000; Sundell and Ylönen 2004), over changes in vigilance and foraging (Brown 1999; Ylönen and Brown 2007; Embar et al. 2011), to drastic changes in the reproductive behaviours (Ylönen and Ronkainen 1994; Sih 1994; Mappes and Ylönen 1997; Mönkkönen et al. 2009; Haapakoski et al. 2012, 2018; Sievert et al. 2019).

If a prey individual survives a direct encounter with a predator, it may increase its own and its conspecifics’ survival and later fitness by signalling predator presence intraspecifically. Several means of intra-species predator communication have been studied in animals, from simple group flight behaviours in birds (Adamo and McKee 2017) to elaborate vocal signalling in primates (Ouattara et al. 2009) and *Mungotinae* (Townsend et al. 2012; Collier et al. 2017). Another pathway of communication is fear or risk signalling body secretions or alarm pheromones (AP). These are widespread in invertebrates, such as sea anemones (Howe and Sheikh 1975), ants (Crewe and Blum 1970b), aphids (Bowers et al. 1972; Beale et al. 2006) or mites (Kuwahara et al. 1989), but also occur in vertebrates such as fish (von Frisch 1938; Wisenden et al. 2004; Mathis and Smith 2008). In the last two decades, a growing number of studies were able to show the presence of AP also in mammals, such as Wistar rats (Kiyokawa et al. 2004; Gutiérrez-García et al. 2007; Inagaki et al. 2009, 2014), C57BL/6J and OMP-GFP mice (Brechbühl et al. 2013), Cabrera voles (*Microtus cabrerae*) (Gomes et al. 2013), and even in domestic cattle (Aubrac breed) (Boissy et al. 1998) and pigs (Vieuille-Thomas and Signoret 1992). Several of the aforementioned species live in social groups, so the secretion of AP serves to warn the group, family or colony.

While the structure of AP remains unresolved for most mammalian species, it has been identified in, for example, aphids (Bowers et al. 1972), sea anemones (Howe and Sheikh 1975), and several insects (Heath and Landolt 1988; Kuwahara et al. 1989). Work on lab rodents has allowed for the analyses of alarm pheromones in Wistar rats (Inagaki et al. 2014), and C57BL/6J and OMP-GFP mice (Brechbühl et al. 2013).

In this study, we use the term “pheromone” to indicate semiochemical communication between individuals of the same species, as opposed to allelochemicals which facilitate communication between two different species (Dicke and Sabelis 1988; Sbarbati and Osculati 2006). We acknowledge that the secretion discussed in this study may have allelochemical properties, but there is no evidence of this in mammals yet.

Semiochemical communication is of great importance in mammals (Müller-Schwarze 1983; Dehnhard 2011; Apps 2013). It is used to convey a wide array of information, among others reproductive status (Pankevich et al. 2004), immunocompetence (Spehr et al. 2006), stress (Gomes et al. 2013) and effects in the mate choice (Roberts et al. 2010). This does not only occur in small mammalian species (Gomes et al. 2013; Inagaki et al. 2014), but also in large ones, e.g. muskox (*Ovibos moschatus*) and giant pandas (*Ailuropoda melanoleuca*) (Flood 1992; Wilson et al. 2018), as well as in primates (Evans 2006; Setchell et al. 2011) and humans (Stern and McClintock 1998; Thornhill and Gangestad 1999).

Previous behavioural studies have already shown alarm pheromone effects on reproductive behaviour in bank voles, specifically differences in the number of offspring (Haapakoski et al. 2018), the amount of parturitions (Sievert et al. 2019), and several transgenerational effects (Sievert et al. 2020). While the effects of an alarm pheromone exposure have been studied, the actual nature remains unclear. This study combines two goals with two different experimental designs: first to identify the chemicals involved in semiochemically signalling alarm in bank voles and second, to verify the effects of these alarm compounds on behavioural decisions of bank voles in the field compared to direct predator presence cue in form of predator odour. In the laboratory study, we sampled vole-derived volatile organic compounds (VOC) after exposing our experimental bank voles to three different stimuli: a live predator (P), handling by a researcher (H), and no stimulus (C). The VOCs were collected by dynamic headspace sampling and analysed by gas chromatography-mass spectrometry (GC–MS). In the field study, we investigated how the presence of alarm pheromone, compared to predator odour and a control, shapes the foraging effort of voles over time. For the field part of the experiment, we predicted alarm pheromones to carry important but sensitive information, and expected to see only short-time effect of volatile APs compared to more long-lasting risk cue of predator odours.

## Materials and methods

### Study animals and site

The bank vole (*Myodes glareolus*) is one of the most common small rodents living in a variety of northern temperate and boreal European forest habitats west of the Urals (Stenseth 1985). The species is granivorous-omnivorous, with their diet consisting mainly of seeds and buds, but also of other plant materials or invertebrates (Hansson 1979; Eccard and Ylönen 2006). In Central Finland, where this work was conducted, bank voles breed three to five times per season, which lasts from May until September (Mappes et al. 1995; Koivula et al. 2003).

Bank voles are preyed upon by a diverse predator assemblage, including least weasels (*Mustela nivalis*) and stoats (*Mustela erminea*) (Ylönen 1989; Meri et al. 2008). The least weasel is an especially effective hunter of voles due to their size and excellent hunting skills, least weasels are likely able to kill bank voles whenever the two species come into direct contact (Tidhar et al. 2007; Haapakoski et al. 2012).

We conducted our study at Konnevesi Research Station in Central Finland (62°37′N, 26°20′E). In the laboratory, males and females were maintained in the same room. The adult voles used in the study were wild-caught individuals that were housed in the lab during the winter months preceding the study period. Winter colonies are formed from the last cohort of voles of the previous summer. Thus, their age at the time of the experiment is about 7 months. The winter population is housed on a short photoperiod (8L:16D) at around 17 °C throughout the winter and male voles’ testes are abdominal and female vaginas are closed. Samples were taken from non-reproductive animals, to minimize contamination related to oestrus cycles or sexual maturity. All animals were individually marked with ear tags (#1005-1L1, National Band & Tag Company, Newport, KY, USA). Voles were kept individually in 42 cm × 26 cm × 15 cm transparent cages with wire mesh lids and supplied with ad libitum water and food. 7 days prior to sampling voles were placed into smaller 24 × 18 × 14 cm cages, equipped with the glass sampling container. The bedding materials in each cage consisted of wood shavings and hay.

Weasels were housed individually in 60 cm × 160 cm × 60 cm cages in an outdoor shelter. Each cage had a nest box and wood shavings and hay as bedding. Throughout the experiment (and during the two-week period before its initiation), weasels were exclusively fed dead bank voles.

### Treatments and VOC sampling

One week before sampling, voles were changed to the small sampling cages containing their usual bedding, including a glass sampling container with a volume of 250 ml covered with a dark cardboard sleeve to simulate a safe refuge. This served to minimize the stress to the vole as much as possible. The control (C) treatment was achieved by switching the glass container for a clean one. The lid to the glass container was closed as soon as the vole entered it voluntarily. Every lid was fashioned with an inlet and outlet and the inside of each lid was covered with a sheet of polytetrafluoroethylene to prevent reactions of the VOC with the lid. Once the lid was attached, the sampling of the air from the chamber to get the control sample was started. The handling (H) treatment consisted of 3 min of simulated standard handling procedures by the same researcher for every sampling (sexing, checking ear tag, checking PIT tag etc.) after which the animals were immediately transferred to a sampling container. For the predator (P) treatment, a vole in a live trap (Ugglan Special, Grahnab AB, Gnosjö, Sweden), was introduced into a weasel cage for 3 min. Afterwards, the vole was directly transferred into the sampling container. Each vole was sampled for VOCs individually.

Containers were cleaned at 75 °C for 20 min with water before and between sampling bouts. Pressurized (Gardner Denver Thomas GmbH, Puchheim, Germany) and filtered, both through an air filter (Wilkerson model M03‐C2‐X00; Wilkerson Corp., Richland, MI, USA) and through active charcoal, inlet air was introduced into glass containers at a flow rate of 255–260 ml min^−1^. After 20 min of flushing air through the tubes and filters, but not the sampling containers, VOC emissions were collected for 20 min (length determined with pilot samples) into pre-conditioned cartridges filled with 200 mg Tenax TA (60/80 mesh, Markes International, UK) positioned at the outlet of the glass container. Cartridges were connected via clean silicone tubes to a vacuum pump (Bühler Technologies GmbH, Ratingen, Germany), which pulled air through the cartridges with a flow rate of 240 ml min^−1^. Inlet and outlet airflows were calibrated with a gas flow calibrator (mini Buck calibrator, Buck, USA).

After collection, cartridges were stored at 4 °C for a maximum of 3 weeks before analysis. Blanks (collected from empty glass containers) were also sampled with the same method to identify potential contaminants. The blanks were collected daily from the room where the VOC collection took place and from inside the weasel cages to exclude a potential contamination of weasel odour in our samples. Analysis of VOCs collected into the cartridges was performed by GC–MS (7890A GC and 5975C VL MSD; Agilent Technologies, USA) with samples thermally desorbed with an automated thermal desorption unit (TD-100; Markes International Ltd, UK). Samples were desorbed at 250 °C for 10 min, and cryofocused at − 30 °C in splitless mode. The column used to separate molecules was an HP5-MS (60 m × 0.25 mm × 0.5 µm, Agilent, USA). The chromatographic program was set up as follows: 40 °C at the start with a hold of 2 min, a 3 °C min^−1^ temperature ramp until 210 °C, and then a 10 °C min^−1^ temperature ramp to 300 °C. This last temperature was held for 5 min to clean the column. The carrier gas was helium. VOC identification was conducted via comparison with a series of analytical standards [see Saunier and Blande (2019)], comparison of mass spectra to the NIST and Wiley libraries and the calculation of Kovats indices (through the injection of alkanes C_8_–C_20_) with comparison to available literature (Adams 2007) (https://webbook.nist.gov/). The following analytical standards were used: 2-hexenal, 3-hexen-1-ol, benzaldehyde, 3-hexen-1-ol acetate, nonanal, benzyl nitrile, methyl salicylate, alpha-pinene, beta-pinene, beta-myrcene, alpha-phellandrene, 3-carene, limonene, eucalyptol, ocimene, linalyl acetate, caryophyllene, bisabolol. Once the identification was done, the quantification for each compound was realized based on calibration curves obtained with the injection of analytical standards used for identification. Then, we normalized the quantity obtained according to the inlet and outlet flows as well as the time of collection (see below).

We provide the experimental *m*/*z* spectra of 2-ocatanone, 1-octanol, and unknown compound 7 (Appendix 1), along with the theoretical NIST spectra for 2-ocatanone and 1-octanol (Appendix 2) in the Supplemental Material.

A total of 23 voles was used in this experiment, 13 for the C treatment and the same ten animals for both H and P.

### Field experiment

Field study was conducted using five 0.25-ha outdoor enclosures close to the Konnevesi Research Station in Central Finland (Ylönen and Eccard 2004) during July and August. Eight voles (four of each sex) were released in five enclosures each. With two repetitions, this resulted in 80 voles total. The enclosures were emptied of other rodents by live trapping before each replication. One week after releasing the voles, three wooden boxes (60 × 40 × 30 cm), with lids, about 10 m apart from each other, were arranged in a triangle at the centre of each enclosure. Each box contained one odour cue, control (C), predator odour (PO) or alarm pheromone (AP). The 1 dl odour cues were obtained as described in Sievert et al. (2020), i.e. clean wood shaving, soiled bedding from weasel cages, and bedding from weasel exposed voles, respectively. Each box contained further a seed tray for determining foraging efficiency of voles under each treatment using the giving-up-density (GUD) method (Brown 1988) (explained in the next paragraph). The trays were lidless boxes (19 × 19 × 6 cm) containing 8 dl of sand into which 20 unhusked sunflower seeds were mixed. The foraging patch was renewed each day, the sand was sieved and the remaining untouched seeds were counted to obtain the GUD.

Brown (1988, 1999) framed the harvest rate an animal makes at a given patch as a balance of the energetic gains and costs attributed to foraging effort, predation, and missed opportunity costs. The density of food remaining in a patch after the forager stops foraging is called a giving‐up density (GUD) (Brown 1999) and reflects the point where the energy remaining in the patch is equal to or outweighed by the combined costs to the forager. The GUD, as a method, has been adapted to test a large variety of elements affecting the strategic decisions animals take (Bedoya-Perez et al. 2013) and has been widely applied as a measure for habitat use (Ylönen et al. 2002; Orrock et al. 2004; Bleicher et al. 2018). In predator–prey studies, a low GUD (more consumed) is interpreted as an indicator of low perceived predation risk, while a high GUD (less consumed) is an indicator of a high perceived predation pressure(Brown 1999; Bedoya-Perez et al. 2013; Bleicher 2017).

### Data analysis

The Emission Rates of VOCs collected by dynamic headspace sampling (ER) were calculated with the following formula:

$${\text{ER}}\user2{~} = ~\frac{{X*Ai}}{{t*Ao}}$$

with ER expressed in ng * h^−1^ * vole^−1^. *X* is the compound quantity (ng), *Ai* and *Ao* are the inlet and outlet air flows (ml * min^−1^), respectively, and *t* is the sampling time in h.

Statistical analyses were performed with the R software (R Core Team 2021). Partial Least Squares Discriminant Analysis (PLS-DA) was performed on ER for all treatments using the package ‘vegan’ (Oksanen et al. 2020) and ‘RVAideMemoire’ (Hervé 2021) with a cross-validation based on 50 submodels (fivefold outer loop and fourfold inner loop). Pairwise tests were performed based on PLS-DA with 999 permutations to highlight the differences between treatments. The PLS-DA graphics were drawn with ‘MetaboAnalystR’ (Chong and Xia 2018; Chong et al. 2019). The Variables Importance for Projection (VIP) scores, obtained through PLS-DA, were used to select the compounds of interest (the ten compounds with the highest scores). Kruskal–Wallis tests followed by Nemenyi post hoc tests were done for these components of interest to compare the ER.

For the GUD measurements, generalized linear mixed models (GLMM) with a Poisson distribution were calculated, ‘lme4’ (Bates et al. 2015). To achieve the best model fit, first the interaction was removed, then other factors, only leaving Treatment for the simplest model. Each treatment was compared to the C (control) treatment. The most fitting model was chosen based on AICc, package ‘MuMIn’ (Barton 2020). A model was considered the best if the difference in AICc from the next model was greater than 2.5. Appropriate random effects were chosen by AICc.

All plots were generated with ‘ggplot2’ (Wickham 2016) and ‘MetaboAnalystR’ (Chong and Xia 2018; Chong et al. 2019).

## Results

### Emission rates

**Fig. 1:**
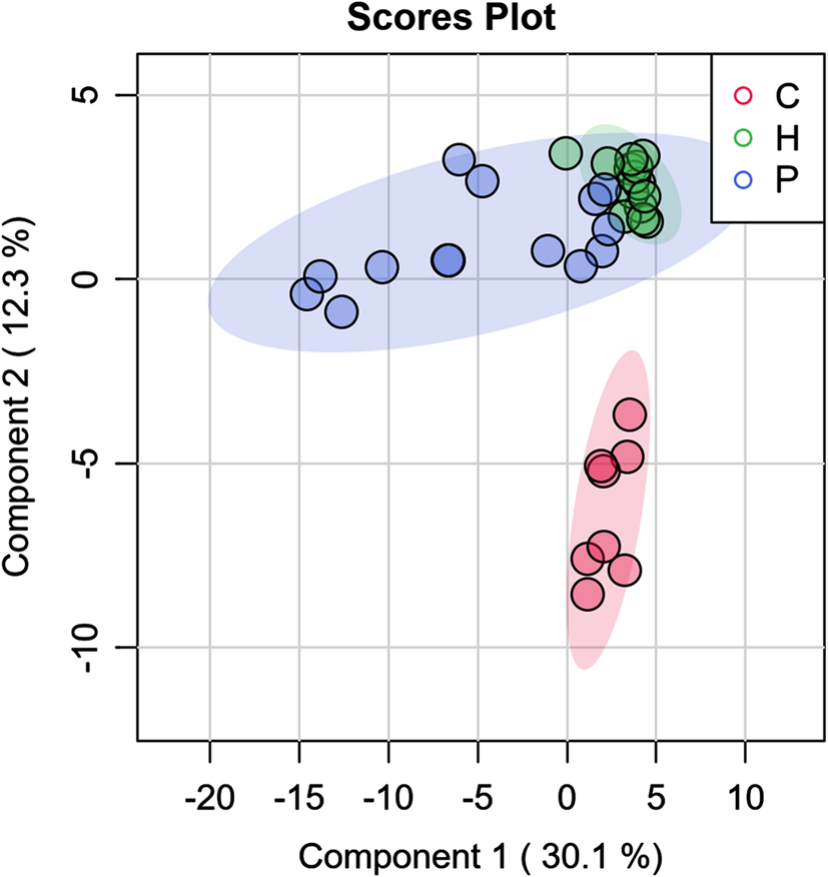
Partial Least Squares—Discriminant Analysis (PLS-DA) based on emission rates according to treatment. Treatments: control (C), Handling (H), and Predator (P)

**Fig. 2:**
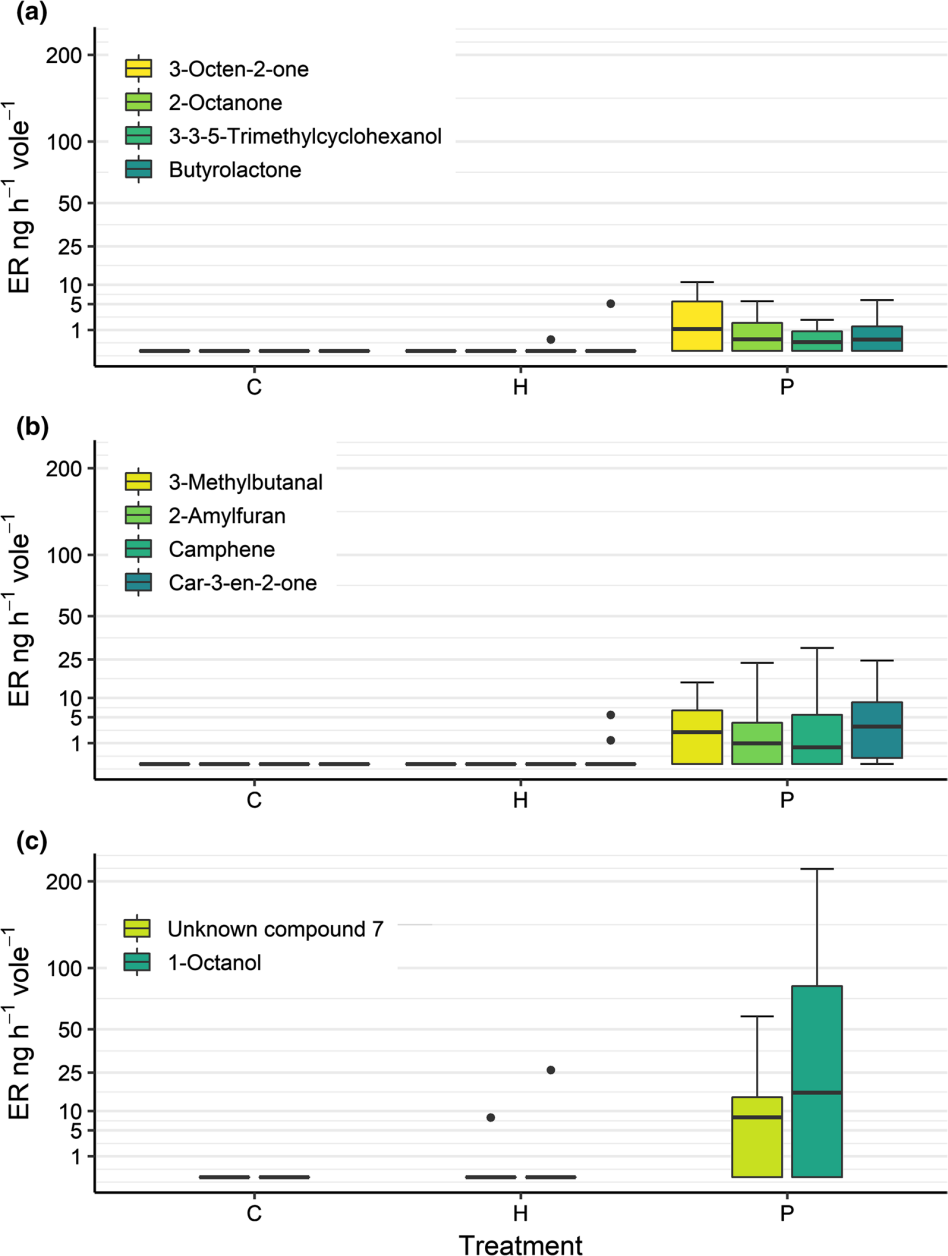
Total emission rates (ng * h^−1^ * vole^−1^) for the compounds of interest, grouped by treatment. Treatments: control (C), Handling (H), and Predator (P). Components in panel **a**, **b** and **c** are grouped by maximum emission rates during the experiment for an easier visual comparison. All components show significant differences between P vs C and P vs H, see Table 1 for details

To investigate differences at the compound level, PLS-DA was performed for the emission rates of the individual compounds emitted for each treatment (Fig. 1). A global permutation test of the PLS-DA showed significant differences (PLS-DA, 999 permutations, *P* = 0.001), while a pairwise permutation test confirmed these (PLS-DA, 999 permutations, *P* = 0.001) for all three pairwise comparisons. An analysis of the ten compounds of interest revealed significantly higher ER in the P treatment compared to both H and C, analysed by a Kruskal–Wallis test (Table 1). None of the ten compounds was detected in the C samples, and five were detected in the H samples at a low rate (Fig. 2). An analysis focusing on sex differences for the ten compounds found no significant differences.

**Table 1:** Top 10 alarm pheromone components, sorted by VIP score

Component	CAS	Retention time (minutes)	VIP score	Difference C–H (*P *value)	Difference C–P (*P *value)	Difference H–P (*P *value)
3-octen-2-one	1669-44-9	23.433	1.897	1	0.005	< 0.001
3-methylbutanal	590-86-3	6.986	1.896	1	0.005	< 0.001
2-amylfuran	3777-69-3	21.031	1.755	1	0.01	0.002
2-octanone	111-13-7	20.957	1.755	1	0.01	0.002
camphene	79-92-5	18.898	1.753	1	0.01	0.002
3-3-5-trimethylcyclohexanol	116-02-9	24.150	1.683	0.95	0.007	0.004
Unknown compound 7	NA	31.087	1.676	0.955	0.006	0.003
1-octanol	111-87-5	25.016	1.674	0.95	0.007	0.004
Car-3-en-2-one	107493-44-7	32.243	1.649	0.861	0.003	0.004
Butyrolactone	96-48-0	16.972	1.624	0.924	0.009	0.007

The CAS identifier together with the retention time is reported for each component. *P *values for the Nemenyi post hoc test for each comparision are shown

### Giving-up-densities

**Fig. 3:**
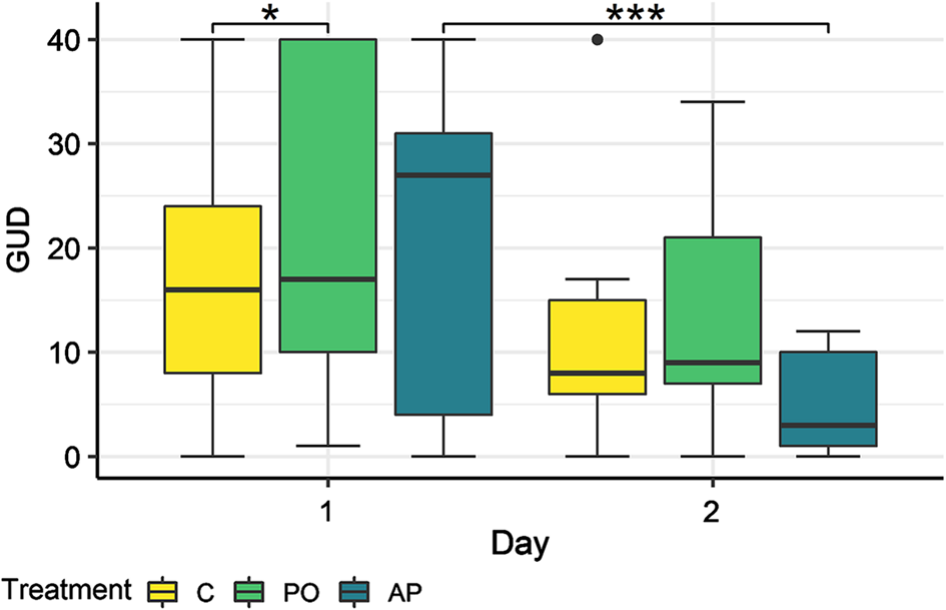
Giving-up density by treatment. Treatments: control (C), predator odour (PO), and alarm pheromone (AP). Asterisk (*) indicates a significant difference from control at *P* < 0.05. Three asterisks (***) in this figure indicate a significant difference from the same treatment on the previous day at *P* < 0.001

The effects of predation risk cue and AP on the GUDs were similar during the first day of the experiment. Voles foraged on average 1.25 seeds less in the PO patch (GLMM, d*f* = 6, *P* = 0.0403) compared to the C patch during the first day (Fig. 3). On the second day (Fig. 3), the voles foraged overall about 30.2% more (GLMM, d*f* = 6, *P* = 0.004) but significantly more, about 74.3% more in the AP patch (GLMM, d*f* = 6, *P* < 0.001).

## Discussion

The first result in the volatile compound (VOC) analyses shows clearly that a disturbed or scared individual smells differently than an undisturbed control vole (Fig. 1). The grouping of the different treatments clearly shows no overlap of the VOCs of animals from the control group and animals from either the handling or weasel exposure group. This simple result verifies the idea that animals can use body odours for signalling and information exchange between conspecifics (Flood 1992; Inagaki et al. 2009; Wilson et al. 2018). The handling and predator-scared groups overlap. However, the range of handling compounds seems to be very narrow compared to the wider range of possible fear compounds.

Further, our study could identify and narrow down the list of possible VOCs, which could act as alarm pheromones in bank voles. We were able to identify ten compounds of interest, which all appear with higher emission rates in animals who previously encountered a weasel (P treatment). We also were able to show that in our field experiment, AP secretion lost their alarming function and efficiency after just one day in the field. It seems that after the volatile alarming compounds vanish, longer lasting social odours are left and, as shown in many studies before, social odours may signal safety (Kiyokawa 2015; Al Aïn et al. 2017) and they could attract voles for non-risky foraging.

From our list of ten compounds of interest, most have been previously found in animals (see Appendix 3. for a list of references) with the exception of Car-3-en-2-one, which to our knowledge has not been found in other animals. Two of them have previously been associated with alarm pheromones or other alarm secretions. 2-Octanone has been found in the alarm secretions of several ant species (Crewe and Blum 1970a; Dumpert 1972; Brand et al. 1989) and lorises (Hagey et al. 2007). 1-Octanol has been found in the alarm secretions of several bee species (Johnson et al. 1985; Collins et al. 1989; Hunt et al. 2003) and stink bugs (Yamashita et al. 2016). 1-Octanol also showed the highest emission rate of all compounds of interest in our experiment (Fig. 2), followed by unknown compound 7. We provide the experimental m/z spectra of 2-ocatanone, 1-octanol, and unknown compound 7, along with the theoretical NIST spectra for 2-ocatanone and 1-octanol in the Supplemental Material.

Evidence of interpreting heterospecific alarm cues is well established, however only in the aquatic environment (Briones-Fourzán et al. 2008; Vogel et al. 2017; Magellan et al. 2019), with the exception of one termite species (Cristaldo et al. 2016). While there is strong evidence that phylogenetic closeness is a major factor (Hazlett and McLay 2005), there is evidence of cross-phyla communication (Kaliszewicz and Uchmański 2009). In terrestrial species, interspecies communication of alarm signals appears most commonly with alarm calls (Templeton and Greene 2007; Vitousek et al. 2007; Lea et al. 2008; Magrath et al. 2009). Within vertebrates, there are examples of the ability to interpret alarm calls correctly across taxonomic classes (Vitousek et al. 2007; Lea et al. 2008).

While our experiment does not provide the data to conclude whether there is a common structure in alarm chemicals, there is evidence from previous work permitting us to entertain the possibility. This would be a potential explanation for the occurrence of our identified compounds in, mostly, insects. In our study, we took only into account the major compounds to highlight potential alarm pheromone. However, we could have missed important signals by choosing this method. Indeed, it has been shown in plant–insect interactions, that minor compounds could have an important effect as chemical cues just like major compounds (Clavijo Mccormick et al. 2014). To go further, a similar experiment should be done focused on minor compounds.

A previous attempt to find a common features of olfactory communication (in terrestrial vertebrates) concluded that the range of compounds is widespread and bigger range of species is needed for proper conclusions (Apps et al. 2015). We share the assessment, as the studies on mammalian alarm pheromones are scarce. Unlike the work by Brechbühl et al. (2013), which found sulphur-containing compounds, our compounds of interest did not include any nitrogen- or sulphur-containing chemicals. This might be partially due to a completely different sampling method. While our method is non-invasive, the work by Brechbühl et al. (2013) included CO_2_ euthanasia to induce stress. Their results have been challenged by (Kiyokawa et al. 2013), pointing out that sampling from sacrificed animals results in collecting early decay volatiles. However, work on rats identified sulphur- or nitrogen-free chemicals as AP (Inagaki et al. 2014). Their work, with methods comparable to ours, identified 4-methylpentanal and hexanal as potential APs, which were not part of our compounds of interest.

While sampling from live animals allows for a greater risk of contaminations, it also allows for more ecologically relevant information. In our experiment, the animals were contained, but similar methods showed the possibility to sample from freely roaming individuals (Weiß et al. 2018). Our methods aimed for a non- or minimal-invasive approach, but also to apply a stimulus, i.e. predator exposure, that is similar to a stimulus in the wild. We believe that the methods in this experiment represent a good balance between a controlled and natural environment.

In our field experiment, no clear difference in foraging effort was observed in the AP GUD was observed on the first day, which is in line with our previous results (Sievert et al. 2019). However, a clear increase in foraging effort in AP patches after just one day, we suggest two factor for explaining this result. First, the AP is very short-lived and the remaining odour just signals the presence of conspecifics, or, secondly, the AP becomes rapidly so diluted that it requires a greater investigation effort (Parsons et al. 2018), which in turn leads to the discovery of food resources in the GUD patches and increased foraging. Either way, the information content concerning a predator presence or risk appears to be minimal at this point. Previous studies on bank vole AP argued that it is secreted in cases of immediate and acute risk (Sievert et al. 2019) and should it have an effective alarming function, it needs a rapid transfer to other conspecifics, group members or even kin. The short-lived character of AP in the field experiment supports this idea.

While weasels are the main factor of vole mortality (Norrdahl and Korpimäki 1995), previous work on vole-weasel interactions has shown that, if presented with the opportunity, bank voles prefer to take arboreal escape routes while chased and weasels are unlikely to follow (Mäkeläinen et al. 2014). This, or other immediate survival enhancing responses, increase the chance for a successful escape and lays the fundament for evolution of adaptive signalling of conspecifics via AP.

To summarize, in this study, we adapted a new method to identify a group of chemicals likely to serve as alarm pheromone compounds in a common mammal species, the bank vole. Three of those, namely 1-octanol, 2-octanone, and unknown compound 7, are likely to be the main actors. In the field experiment, we confirmed that the information carried in AP is short-lived, as we were expecting if AP functions to signal an acute and rapid event of very high risk. Our result expands the knowledge on predator–prey interactions and how predation risk can be communicated to unaware conspecifics.

## Supplementary Information

Below is the link to the electronic supplementary material.

Supplementary file1 (XLSX 41 KB)

Supplementary file2 (DOCX 93 KB)

Supplementary file3 (XLSX 19 KB)

## Data Availability

The R code is available from the figshare repository at https://doi.org/10.6084/m9.figshare.13148465 and the raw data is available from the figshare repository at https://doi.org/10.6084/m9.figshare.13148351.

## Code availability

The R code is available from the figshare repository at https://doi.org/10.6084/m9.figshare.13148465.
