# Supplementary material for: Bank vole alarm pheromone chemistry and effects in the field
Source: Oecologia. 2021 Jun 25;196(3):667–77. doi: 10.1007/s00442-021-04977-w (PMC8292297; doi:10.1007/s00442-021-04977-w)
Supplement: Supplementary file 2 — Supplementary file2 (DOCX 93 KB) [file 442_2021_4977_MOESM2_ESM.docx]

Bank vole alarm pheromone chemistry and effects in the field

# Appendix 1


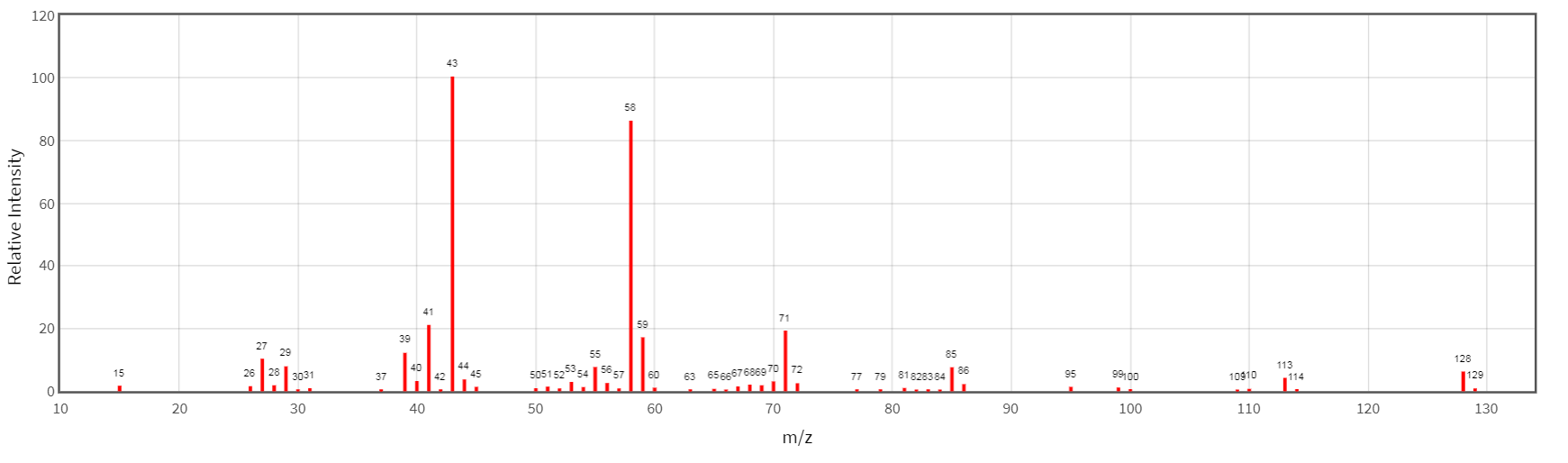


Figure S1: Theoretical mass spectrum for 2-octanol


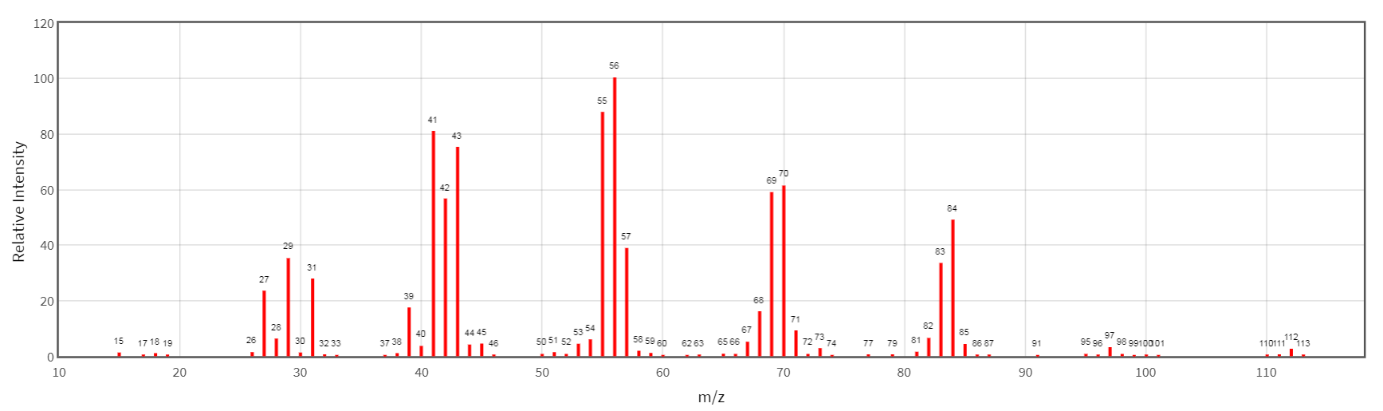


Figure S2: Theoretical mass spectrum of 1-octanol
